# Exploring barriers to primary care for migrants in Greece in times of austerity: Perspectives of service providers

**DOI:** 10.1080/13814788.2017.1307336

**Published:** 2017-04-07

**Authors:** Maria Papadakaki, Christos Lionis, Aristoula Saridaki, Christopher Dowrick, Tomas de Brún, Mary O’Reilly-de Brún, Catherine A O’Donnell, Nicola Burns, Evelyn van Weel-Baumgarten, Maria van den Muijsenbergh, Wolfgang Spiegel, Anne MacFarlane

**Affiliations:** ^a^ Faculty of Medicine, University of CreteHeraklionGreece; ^b^ Department of Social Work, School of Health and Social Welfare, Technological Educational Institute of CreteHeraklionGreece; ^c^ Institute of Psychology, Health and Society, University of LiverpoolLiverpoolUK; ^d^ Discipline of General Practice, School of Medicine, National University of IrelandGalwayIreland; ^e^ General Practice and Primary Care, Institute of Health & Wellbeing, College of Medical, Veterinary and Life Sciences, University of GlasgowGlasgowUK; ^f^ Lancaster Medical School, Faculty of Health and Medicine, Furness College, Lancaster UniversityLancasterUK; ^g^ Department of Primary and Community Care Radboud University Medical CenterNijmegen The Netherlands; ^h^ Pharos Centre of Expertise on Health DisparitiesUtrechtThe Netherlands; ^i^ Centre for Public Health, Medical University of ViennaViennaAustria; ^j^ Graduate Entry Medical School, University of LimerickLimerickIreland

**Keywords:** Migrants, primary healthcare, capacity, attitudes, financial crisis

## Abstract

**Background:** Migration in Europe is increasing at an unprecedented rate. There is an urgent need to develop ‘migrant-sensitive healthcare systems’. However, there are many barriers to healthcare for migrants. Despite Greece’s recent, significant experiences of inward migration during a period of economic austerity, little is known about Greek primary care service providers’ experiences of delivering care to migrants.

**Objectives:** To identify service providers’ views on the barriers to migrant healthcare.

**Methods:** Qualitative study involving six participatory learning and action (PLA) focus group sessions with nine service providers. Data generation was informed by normalization process theory (NPT). Thematic analysis was applied to identify barriers to efficient migrant healthcare.

**Results:** Three main provider and system-related barriers emerged: (a) emphasis on major challenges in healthcare provision, (b) low perceived control and effectiveness to support migrant healthcare, and (c) attention to impoverished local population.

**Conclusion:** The study identified major provider and system-related barriers in the provision of primary healthcare to migrants. It is important for the healthcare system in Greece to provide appropriate supports for communication in cross-cultural consultations for its diversifying population.

Key messages

- Discriminatory attitudes and other provider and system-related barriers are evident in the provision of primary healthcare to migrants in Greece.
- Providers feel unable to fulfil their role efficiently under limited system support and contribution to decision making.
- Training and guidelines promoting cultural competence are necessary in the Greek primary healthcare.

## Introduction

Equity in access to health services has been a fundamental objective for many European health systems, including Greece [1,2]. As part of this objective, the need to develop ‘migrant-sensitive healthcare systems’ has been raised as a key issue by global organizations [3,4]. Primary care is ideally placed to address the inequities and challenges apparent in the provision of healthcare for migrants and primary care providers are often acutely aware of the social circumstances in which people live [5,6].

Greece has no comprehensive policy regarding migrants’ access and use of healthcare services, despite the high influx of refugees and migrants evident during recent years [7]. The basis of healthcare entitlement is a mix of tax, social insurance, private and out-of-pocket payments and this applies to migrants that legally reside in the country. Undocumented migrants are only to receive emergency care and are not entitled to health insurance [8].

Not surprisingly, Greece’s migrant integration policy index (MIPEX) score for the health of migrants was unfavourable, revealing the limited available services and high out-of-pocket payments [9].

In daily practice, many general practitioners (GPs) at primary healthcare clinics have been serving as gatekeepers for both documented and undocumented migrants and this seems to have placed a high burden to primary care service providers [10]. The recent financial crisis and the austerity measures have exacerbated this problem resulting in a dysfunctional primary healthcare sector with many cutbacks in healthcare services to vulnerable groups [11–15]. However, there has been no analysis of service providers’ knowledge and experience of delivering care to migrant populations. This paper focuses on the question ‘what are Greek primary care providers’ perspectives on barriers to healthcare for migrants?’

This analysis is part of a larger European project (RESTORE) involving five European countries (Austria, Greece, Ireland, Netherlands, and UK). RESTORE focused on the implementation journeys of guidelines and training initiatives that are designed to support communication in cross-cultural primary care consultations and provided an opportunity for analysis of Greek service providers’ perspectives on migrant healthcare [16,17].

## Methods

### Study design

RESTORE is a qualitative case study based on a unique combination of qualitative methodology—participatory learning and action (PLA) and contemporary social theory—normalization process theory (NPT) [16]. PLA is a practical and active approach to enable different groups and individuals to collaborate jointly to form a decision in a democratic way [18]. The iterative and organic nature of PLA encourages diverse stakeholders to engage in cycles of research, co-analysis, reflection and evaluation over time. NPT is a contemporary social theory, which provides a conceptual framework to investigate and support the implementation of interventions into daily routine [19]. PLA and NPT are described in more detail in a separate paper [20].

The Bio-ethical Committee of the University Hospital in Heraklion Crete approved the study with protocol number 8297/19-7-2010.

### Selection of study subjects

As per our study protocol, the sample was developed following the principles of purposive sampling and sought participation of multiple stakeholders with major involvement in planning and delivery of migrant healthcare [16,21]. The focus of this paper is placed on primary care providers only and the views of migrants and other stakeholders are reported elsewhere [21]

Healthcare centres with high numbers of migrant users operating in two prefectures of the Cretan region in Greece (Heraklion, Rethymnon) were invited to be involved in the study via their representative primary care providers. Nine service providers participated in the study representing two groups of primary care providers; GPs and nurses (Table 1).

**Table 1.** Participants’ profile.

	*n*
Gender	*n*
Men	3
Women	6
Age, years	
18–30	1
31–54	7
55+	1
Nationality/ethnicity	
Greek	8
Dutch	1
Stakeholder group	
Primary care doctors	5
Primary care nurses	4

### Qualitative methods

Participants were invited to participate in a series of six, mixed stakeholder PLA-style focus group discussions, which have worked well in previous participatory migrant health projects [22]. The focus groups were conducted within the second half of 2013 and were facilitated by two moderators (MP, AS), experienced qualitative researchers who had received extensive training in the use of PLA and NPT as part of the RESTORE project. The focus group meetings involved all the nine service providers with the exception of two meetings that involved six and seven participants respectively. If a participant missed a focus group meeting, they caught up with the discussion from the PLA commentary charts (Table 2), which is a technique that captures a visual record of all key messages, which can be brought to subsequent focus groups to ensure that all participants are aware of the emergent data.

**Table 2.** PLA techniques used in the focus group discussions.

Commentary charts	Team-generated records of stakeholders’ discussions about the guideline separated into three categories: positive aspects, negative aspects and questions to be checked out.
Flexible brainstorming	Fast and creative approach of using materials to generate information and ideas about the topic.
Direct ranking	A transparent and democratic process that enables a group of stakeholders to indicate priorities or preferences, by ranking the guideline as ‘most suitable’ to ‘least suitable’ for implementation in RESTORE.

The focus group discussions were facilitated using a topic guide based on the NPT theory (Box 1), which explored participants’ views on the individual and organizational barriers as well as the implementation challenges of a set of five guidelines and training initiatives (G/TIs), which were designed to address the language and cultural barriers in cross-cultural consultation. These G/TIs were identified earlier in the project, at another stage of fieldwork as being suitable for the Greek setting [23].

Box 1 .Normalization process theory (NPT)—based items included in the focus group guide.

1.Does this guideline make sense?
2.What impact will the implementation of this guideline have in the Greek primary care setting?
3.Does the guideline fit into the local priorities of the primary healthcare setting?
4.Are you willing to engage and contribute to the adaptation of this guideline?
5.Are you willing to participate in the implementation of this guideline?
6.What barriers do you see on an individual level to implementing this guideline?
7.What barriers do you see on an organizational level to implementing this guideline?
8.Are there people that are willing to drive this guideline implementation forwards?
9.Is it worthwhile to invest time in this guideline implementation?
10.Will this guideline implementation change your existing work practices?
11.Do we have the available resources to implement this guideline?

The PLA-style focus group discussions were tape-recorded and transcribed verbatim for analysis.

### Outcomes and analysis

For RESTORE, thematic analysis of qualitative data was deductive using NPT as our conceptual framework. For the purpose of this paper, thematic analysis was used through an inductive approach [24]. The raw transcripts generated in the RESTORE focus group discussions were analysed anew to answer the research question ‘what are Greek primary care providers’ perspectives on barriers to healthcare for migrants?’ More specifically, a case description was initially drafted for each of the six PLA focus group discussions using all data. Then, the process included the coding of data into meaningful groups and establishing a coding scheme. Two persons coded the data independently (MP, AS). The list of different codes were sorted into potential themes regarding the barriers encountered by healthcare professionals in primary care delivery to migrants, based on recurring regularities and coherent patterns of meaning [24].

## Results

### Study population

Detailed information of the study participants are shown in Table 1. Most participants were women (*n* = 6), aged between 31 and 55 years (*n* = 7) as well as of Greek origin/nationality (*n* = 8). A Dutch healthcare professional, serving the national healthcare system, was included among the study participants.

### Study outcomes

The inductive thematic analysis identified three main themes about barriers in migrant healthcare: (a) emphasis on major challenges in healthcare provision, (b) low perceived control and effectiveness to support migrant healthcare, and (c) attention to impoverished local population.

### Emphasis on major challenges in healthcare provision

Participants acknowledged the sustainability of the healthcare system, which is currently threatened by the financial crisis, as an issue of higher priority as compared with the needs of one particular group of primary care users such as migrants. They referred to a decaying Greek healthcare system, which is currently operating under limited resources and is unable to meet the increased demands in healthcare. They further referred to difficulties relevant to the regular and continuous access to the healthcare system of vulnerable groups of the population such as the uninsured and those with chronic diseases. Most importantly, they underlined their concern about the rapid societal changes and the increase of the unemployed and uninsured population, which they expected soon to have a huge impact on public health and the healthcare sector (Table 3).

**Table 3.** Quotes under the three main themes.

Theme 1. Emphasis on major challenges in healthcare provision			
Quote 1 *What we care about right now is the shortage of equipment and staff not the language difficulties that migrants face….* (GP7)	Quote 2 *It doesn’t matter if they are migrants or Greeks … all patients are underserved and the whole healthcare system is at risk…* (GP4)	Quote 3 *If a migrant is unable to speak in our native language then it is their responsibility to bring an interpreter with them, at our health centre we are so understaffed; we don’t have the time to worry about the migrant that cannot speak Greek….* (GP1)	Quote 4 *I am not sure if such initiatives (introducing interpreting services) are doable …. there are people with chronic diseases that are not eligible of treatment and medication anymore!!* (PHC Nurse 1)
Theme 2. Low perceived control and effectiveness to support migrant healthcare			
Quote 1 *… (a guideline to support cross-cultural consultation) is extremely difficult to implement in Greece, as we do not have registered interpreters for any healthcare setting….* (PHC nurse 3).	Quote 2 *… people from certain migrant groups do not speak Greek fluently and we don’t have the language skills or the time to understand them … we try to do our best with the contribution of the people that accompany them … [as informal interpreters].* (GP1)	Quote 3 *… this is important and necessary in primary care (to introduce interpreters) but seems very difficult, if not impossible, to bring policy changes at this time (of the financial crisis)….* (GP4)	Quote 4 *We are going through a difficult time in Greece now and we are doubtful if this (introducing interpreting services) will be accepted (by central healthcare authorities) as migrants are not a priority….* (GP2)
Theme 3. Attention to impoverished local population			
Quote 1 *… there are many Greek families starving to death while migrants enjoy great privileges as a result of their status ….* (GP1)	Quote 2 *… if I had a migrant and a Greek in the patients’ list I would give priority to the Greek…. they are suffering a lot and they deserve to be treated first in their own country.* (PHC nurse 2)	Quote 3 *I have so many poor people in my patient list … I don’t know any more who is most in need … the only thing I know is that many Greek people can’t even buy their medication anymore and this is the most important right now ….* (GP7)

Box 1 Normalization process theory (NPT)—based items included in the focus group guide.

1. Does this guideline make sense?
2. What impact will the implementation of this guideline have in the Greek primary care setting?
3. Does the guideline fit into the local priorities of the primary healthcare setting?
4. Are you willing to engage and contribute to the adaptation of this guideline?
5. Are you willing to participate in the implementation of this guideline?
6. What barriers do you see on an individual level to implementing this guideline?
7. What barriers do you see on an organizational level to implementing this guideline?
8. Are there people that are willing to drive this guideline implementation forwards?
9. Is it worthwhile to invest time in this guideline implementation?
10. Will this guideline implementation change your existing work practices?
11. Do we have the available resources to implement this guideline?

### Low perceived control and effectiveness to support migrant healthcare

The healthcare providers felt powerless about supporting migrant healthcare with such low capacity in the system. They felt that they were ineffective with regard to their ability to bring changes to the system to improve migrant healthcare. They thought themselves as being the final recipients of political decisions without any scope for active participation in these decision-making processes. They referred to continuous updates to Greek laws and policies regarding migrants’ healthcare and reported a huge difficulty in daily scheduling or in making plans in a healthcare system that keeps changing day-by-day.

Service providers also emphasized their lack of training and skills for working in cross-cultural consultations as significant barriers in the management of language differences in consultations with migrants. They reiterated the resource problem: effective cross-cultural communication is not easy to achieve in a system that lacks resources to enable the development of a culturally competent workforce (see quotes in Table 3).

### Attention to the impoverished local population

Many participants expressed their sympathy and a high concern for the newly, poverty-stricken indigenous Greek population. They strongly emphasized their emerging healthcare needs due to the financial crisis and austerity measures. They discussed the increasing number of uninsured people in Greece, who were experiencing difficulties accessing medical and pharmaceutical care. They underlined the need to pay more attention to these newly vulnerable groups of Greek patients. In some cases, service providers expressed their intention to prioritise the vulnerable Greek population over migrants (see Table 3).

## Discussion

### Main findings

This analysis revealed major provider and system-related barriers in the provision of primary healthcare to migrants in two prefectures of the region of Crete, Greece. At provider level, feelings of powerlessness and unfavourable attitudes towards migrants, combined with the lack of cultural competence were identified as major barriers in healthcare provision to migrant patients. At the system level, austerity measures have led to very limited resources, there is low capacity in the entire healthcare system, which is affecting many Greek people as well as migrants, and there are rapidly changing laws and policies about migrants’ entitlements to healthcare. Some primary care providers report that they would prioritize healthcare for newly, impoverished Greek nationals over migrants.

### System support and the financial crisis

It was not surprising that service providers acknowledged barriers related to the healthcare system and its limited capacity to support migrant patients. It has been noted already that wider austerity measures and an increasingly hostile political climate at the supra-national levels have been shown to have an impact on care [25,14]. In fact, Greece is currently operating under limited resources with 40% cuts in hospital budgets, understaffing, occasional shortages of medical supplies and bribes given to medical staff to jump queues in overstretched hospitals [11]. Besides that, the Greek primary care system is one of the weak ones in Europe not only due to the limited number of GPs per head of the population but due to a number of factors relevant to the organization of the healthcare system and the patients’ access [8].

### Power and contribution to decision making

Service providers expressed concerns about their ability to fulfil their role and duties efficiently in a severely limited healthcare system support and with minimal scope to contribute to national policy level decision making. This concurs with previous research in Greece. For example, general practitioners have publically raised their concerns about the provision of care for migrants and the limited role that they have to address this problem during the financial crisis [26]. Another study found that Greece is struggling with the financial crisis with government-controlled measures to protect public health without the proper design and consensus with stakeholders [27]. At a broader level, primary care providers, particularly GPs, are still seeking full recognition in the Greek healthcare system, which, arguably, compounds these feelings of powerlessness [12,28].

### Attitudes and professional judgement

What is probably most interesting among the results of the current study is the fact that health service providers emphasized their sympathy for a certain group of patients i.e. newly impoverished Greek patients. This could indicate a biased judgement in favour of certain patients, which is in contrast with the universal nature of the public healthcare system. This finding is in line with previous research on Crete, Greece, which indicated a growing societal resistance towards undocumented migrants, as well as a tendency of some GPs to place a higher priority on addressing the health burden of the Greek population as compared with similar health problems of the migrants [10]. This was particularly evident in Teunissen et al.’s study [10], which found that GPs’ were disregarding the primary care system regulations in an attempt to serve undocumented migrants and offer them free and unrestricted access to healthcare. This conflicting evidence needs further research. It also highlights the need to offer primary care service providers with professional guidance and support in dealing with conflicting emotions and professional dilemmas generated at times of political uncertainty and low capacity in the healthcare system.

### Strengths and limitations

We were able to gain reliable data on sensitive topics and we consider this as one of the strengths of this study. The fieldwork and analysis was led by experienced qualitative researchers and complied with good practice in terms of sampling, data-generation and analysis. In addition, our fieldwork was conducted as part of a larger study, which is supported by the use of theory. These findings will be used to advance our knowledge of the inter-relationships between austerity and professional attitudes and practices on implementation processes.

There are certain limitations that need to be mentioned. First, the small number of participants in the study restricts generalizability of current findings. Second, the participants were drawn from one region of Greece and, thus, we cannot claim that the findings apply to other parts of the country. Third, the service providers did not maintain a consistent contribution to all the PLA sessions, implying that the voice of some participants was missing from certain discussions, although our use of PLA Commentary Charts alleviated this in an effective manner. Fourth, we need to acknowledge the fact that the data for this study were collected in 2013 and that the findings reflect a situation evident during that particular period. The austerity measures and the nature and scale of inward migration to Greece in fact have worsened since these data were collected. Last, we acknowledge the potential of social attrition as a source of bias introduced by the researchers in the study. To reduce this bias, we have taken certain measures such as using experienced researchers with different scientific backgrounds, as well as regular data analysis meetings in the Greek team and with the wider consortium throughout the analysis to enhance discussion and debate about the data and our interpretation of them.

### Implications for clinical practice, education, policy or research

The study has identified a number of barriers that seem to hamper the ability of service providers operating in two prefectures of Crete, to respond to migrant patients. Addressing potentially discriminatory attitudes toward migrants, and providing support for primary care providers who are dealing with dilemmas about the growing health inequities among Greek and migrant populations are now needed more than ever.

Guidelines promoting cultural competence also deserve more attention in the Greek primary healthcare system.

A vocational programme incorporating training for GPs and the primary care team on migrant and refugees’ healthcare and on other vulnerable populations in Greece is recommended. Most importantly, this research is timely, as the Greek government is discussing primary healthcare reform and migrant as well as refugee healthcare policy and its results could influence these policy changes.

## Conclusion

The current study has revealed major barriers to primary care for migrants in Greece at the provider level and at the system’s level. Combined efforts are required by the central healthcare authorities, the educational institutes and other key actors in the health sector, such as primary care providers and migrants, to address these barriers so that Greece can move towards a healthcare system that can provide appropriate support for communication in cross-cultural consultations for its diversifying population.
